# Motivational interviewing with American Indian mothers to prevent early childhood caries: study design and methodology of a randomized control trial

**DOI:** 10.1186/1745-6215-15-125

**Published:** 2014-04-14

**Authors:** Terrence Batliner, Karen A Fehringer, Tamanna Tiwari, William G Henderson, Anne Wilson, Angela G Brega, Judith Albino

**Affiliations:** 1 Centers for American Indian and Alaska Native Health, Colorado School of Public Health, University of Colorado Anschutz Medical Campus, Mail Stop F800, 13055 E. 17th Avenue, Aurora, CO 80045, USA; 2 Health Outcomes Program, University of Colorado Anschutz Medical Campus, Mail Stop F443, 13199 E. Montview Blvd., Suite 300, Room 342, Aurora, CO 80045, USA; 3 School of Dental Medicine, University of Colorado Anschutz Medical Campus, 13123 East 16th Avenue, B240, Aurora, CO 80045, USA

**Keywords:** Early Childhood Caries, Motivational Interviewing, Oral Health Behavior Change, American Indian, Community-Based Participatory Research

## Abstract

**Background:**

This randomized control trial assesses the effectiveness of motivational interviewing (MI) to encourage behavior change in new mothers relating to caries prevention when caring for their newborn American Indian (AI) infants and young AI children.

**Methods/Design:**

The study is a randomized control trial. We hypothesize that when motivational interviewing is added to enhanced community oral health education services, the new mothers will achieve greater reduction of caries experience in their AI children compared to those who are receiving enhanced community services (ECS) alone. Six hundred mothers or caregivers of AI newborns will be enrolled into the study and randomized to one of the two intervention groups over a two-year period. The children will be followed until the child’s third birthday. A cost analysis of the study is being conducted in tandem with the enhanced community services, motivational interviewing behavioral interventions, and the dental screenings for the length of the study.

**Discussion:**

The trial is now in the implementation phase and a number of threats to successful completion, such as recruitment and retention challenges in a vast, rural geographic area, have been addressed. The protocol provides a unique model for oral health interventions using principles of community-based participatory research and is currently on schedule to meet study objectives. If the study is successful, motivational interviewing intervention can be applied in AI communities to reduce ECC disparities in this disadvantaged population, with study of further applicability in other populations and settings.

**Trial registration:**

ClinicalTrials.gov, NCT01116726.

## Background

The extensive health disparities experienced by American Indians and Alaska Natives (AI/AN) include oral diseases, such as dental caries (cavities) and periodontal (gum) disease. The United States prevalence of early childhood caries (ECC) is highest in American Indian (AI) children [1]. ECC is defined as the presence of one or more decayed, missing, or filled tooth surfaces in any primary tooth in a preschool-age child between birth and six years of age [2]. A recent study of oral disease on the Pine Ridge Reservation revealed that 84% of children (n = 157) had untreated decay [3]. Early childhood caries in young children often signal the beginning of a downward spiral of increasingly poor oral health and oral health-related quality of life. A cariogenic diet and poor oral health behaviors in the first two years of life are associated with ECC.

Results from the 1991 and 1999 Indian Health Service (IHS) Oral Health Surveys, and more recent studies, indicate that ECC continues to increase in AI/AN children [1]. When compared to other children aged two to five years, the percentage of untreated decay is more than three times higher in AI/AN children (18.7% versus 68%) [1]. An oral health surveillance system for children aged one to five years (n = 8461) was initiated by the IHS in 2010. Overall, 54% of AI children had decay experience, 39% of children had untreated decay, and 36% needed urgent dental care. The prevalence of caries increased with age, with 21% of one-year-olds and 75% of five-year-olds having a history of dental caries [4]. The etiology of ECC is multifactorial and determinants include social, behavioral, biological, and service system factors [5-9]. Behaviorally–mediated risk factors which include cariogenic diet, poor oral hygiene, and unattended bottle feedings in the first two years of life are all associated with ECC [10,11]. These oral health behaviors are related to inappropriate information, a belief that baby teeth are not important, mothers’ previous negative experiences with oral health care, and difficult experiences brushing children’s teeth [12-14].

Motivational interviewing (MI) is a behavioral intervention approach that was first developed for use with alcohol and substance abuse populations [15]. More recent applications have included use in a broad array of health challenges, including oral health. It involves informal assessment by the interventionist of the focal person’s stage of ‘readiness for change’ with regard to a targeted behavior [16]. This collaborative, client-oriented approach supports participants in openly discussing concerns about, and obstacles to, changing behavior. In contrast to more direct approaches in which individuals are told how to change their behavior, MI uses techniques that elicit self-motivating statements and place the responsibility for change directly under the client’s control [17]. This method is based on the principle that exploring and resolving ambivalence about taking action is an essential component in this preparation for change [18-21]. A Scottish trial suggested that actions as simple as formulating an action plan with the focal participant in about one minute can generate significant changes in behavior [22]. Another study reported that even one session of this intervention (with telephone follow-up) increased the use of fluoride varnish and consequently reduced ECC [23].

This manuscript describes the protocol for a randomized control trial to use motivational interviewing (MI) to affect caries prevention behaviors by new mothers of AI infants and young children. The trial is being conducted by the Center for Native Oral Health Research (CNOHR) at the University of Colorado Anschutz Medical Campus (United States), which was established in 2008. It is one of five oral health disparities centers nationally, and one of three Collaborating Centers for Early Childhood Caries (other ECC centers are located at the University of California, San Francisco and Boston University).

## Methods/Design

### Study design

This study is a randomized controlled trial to determine whether behavioral intervention using motivational interviewing with AI new mothers will achieve a greater reduction of caries experience in children younger than three years than enhanced community services alone. Six hundred mothers (n = 600) or caregivers of newborns will be enrolled and randomized to one of two groups over a two-year period with a period of follow-up lasting until the child’s third birthday.

The control group will receive enhanced community oral health services only and the intervention group will receive MI plus enhanced community services. The MI intervention will consist of four visits, the first shortly after childbirth, and again when the children attain 6, 12, and 18 months of age. Enhanced community services includes the provision of oral health supplies for the child and immediate family members, informational brochures with culturally specific artwork and designs, public service announcements in the newspaper and on the tribal radio, and billboards placed in strategic locations on the reservation focusing on the important ECC risk factors. Each of the two intervention groups receives an equal number of visits across the three years of study participation. All study participants will receive financial compensation in the form of a gift card for each study session completed.

The primary outcome will be caries experience, assessed as decayed, missing, and filled tooth surfaces (dmfs) of the children at one, two and three years of age. Secondary outcomes will include caries patterns, costs, dental knowledge, attitudes, and oral health-related behaviors of children and mothers or caregivers assessed at baseline, one, two, and three years.

### Conceptual framework

**Figure 1:**
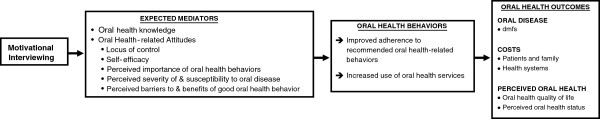
**Conceptual framework for motivational interviewing and oral health outcomes.** The figure presents the conceptual framework that guides the study. This framework will guide the intervention that will impact the oral health behaviors and ultimately the oral health outcomes. dmfs, decayed, missing and filled tooth surfaces.

Figure 1 presents the conceptual framework that guides the study. The framework combines key variables specified in health behavior theory with constructs shown in the literature to be influenced by MI. Each session is conducted within the ‘spirit of MI’, which means the session is collaborative, calls forth or evokes information and motivation from within the participant, and honors the patient’s autonomy to decide what is best for her or him. We hypothesize that MI will lead to positive behavior change (increased adherence to recommended behaviors and use of preventive care), resulting in improved oral health outcomes for the target child. Outcomes of interest include caries experience (as measured by dmfs) and perceived oral health status, as well as reports of changed oral health behaviors, and health care costs associated with dental treatment. Based on prior research examining the impact of MI, we expect that the intervention will result in improved oral health behavior through improved oral health knowledge, self-efficacy, and an increased sense of the importance of good oral hygiene. We theorize that when participants learn more about oral health during the MI sessions, they will be able to formulate oral health care goals for their children; develop the confidence and motivation for behavior change through this supportive discussions about the importance of oral health practices and formulate action steps [24]. The participant will talk of her desires, abilities, reasons, and need for change (DARN), and is likely to arrive at a commitment to change and develop an action plan [24,25]. Socio-demographic characteristics (such as age, education, income) will be recorded so that we can control for the potentially confounding influence of these variables. We will also collect data on a number of variables that may moderate the impact of the intervention.

### Objectives

There are three specific aims of the study. The first is to collaborate with AI community service providers and residents to develop culturally appropriate educational and health promotional materials (brochures, public service announcements aired on the reservation radio station, and billboards) to emphasize the value of family oral health from birth. The second is to create a culturally specific manual for an MI intervention on oral health focusing on mothers or caregivers and their newborn and young AI children. The third is to compare the effectiveness of enhanced community services plus this MI intervention versus enhanced community services alone on the children’s dmfs measures at years one, two and three.

The secondary aims for this study are: to assess other outcome measures, including specific caries patterns and costs of the interventions; and to investigate potential moderators and mediators of the intervention conditions.

### Participants

Study participants are enrolled at the Pine Ridge IHS hospital, powwows, health fairs, local community events, and in Rapid City, South Dakota (United States).

### Inclusion criteria

The participating child must be AI, as defined by the tribe, living on or near Pine Ridge Reservation in South Dakota. The adult must be the mother or caregiver of the newborn AI child, be between 15 and 65 years of age (in the case of minors who are 15 to 17 years of age, consent also is obtained from a parent or legal guardian according to Tribal, State, and IHS rules and regulations), be able to understand and sign a consent form, and be willing and able to follow study procedures and instructions. Mothers or caregivers of twins are included, but only one child will be included in the study, as selected by the mother or caregiver. Fathers are included as members of the larger family setting, but are not the focal point of the intervention. In situations where the father is the primary caregiver; he and his child are eligible for the study.

### Exclusion criteria

Any persons who are not mothers or caregivers of AI children, persons without responsibility to care for a newborn AI child, and parents or caregivers under the age of 15 or over the age 65 are not eligible for this research study. Newborns with serious congenital syndromes expected to adversely affect the development of primary teeth are excluded. This does not include children with cleft lip or palate; these children are not automatically excluded. Participants are also excluded if they cannot understand and sign a consent form, and are not able to follow study procedures and instructions.

### Study site

The trial is being conducted on the Pine Ridge Reservation of the Oglala Sioux Tribe (OST), the second largest reservation in the United States, covering an area of 4,353 square miles. Approximately 32,000 people live on the reservation, often in small, isolated communities and rural clusters. The Pine Ridge Reservation has an unemployment rate of 89% which leads to marked poverty [26]. Three Indian Health Service (IHS) dental clinics are located on the reservation, however, due to high levels of acute need professional dental staff must address these acute problems, leaving little time and few personnel to focus on prevention. The Pine Ridge Reservation was selected as the site for this study because of the high need for oral disease prevention [3], the university’s long standing working relationship with the tribe, and because it is one of the few reservations where the number of births is large enough to allow us to meet statistical power requirements for a trial of this type [27].

### Intervention

#### Motivational interviewing (MI)

A culturally specific MI script was developed with a Native MI consultant, a training manual was compiled, intervention providers were trained, and a pilot test was completed before the MI intervention was used in the field. The intervention providers are local people with some education beyond high school, no oral health training or experience but with experience working in a position with public interaction. The two day training for the intervention providers includes MI material written by the Native MI expert with emphasis on integrating AI and western practices providing the foundation for MI with AI populations [28]. They learn and demonstrate the use of communication skills to work with people as partners as they consider personal behavior change. They practice fundamental MI techniques that focus on helping clients strengthen the motivation to improve oral health behaviors. During training, the intervention providers conduct five audio-recorded practice sessions of mothers or caregivers for review and feedback by the MI intervention director in consultation with the MI consultant. Once the intervention providers are fully trained they will start intervention visits. However, every intervention will be recorded and a fidelity measurement will be conducted (discussed below).

Participants will choose the location where they meet with the MI intervention provider. A participant may choose to meet at their home, in a common public place (such as a local library in a private room), at the field office, or another community program location. Whenever possible, the same intervention provider will meet with the same study participant for each of the four interventions. At each MI intervention visit, the date of the visit, age of the child, content selected for discussion, the mother or caregiver’s decisions regarding next steps, their satisfaction with the visit, and oral health supplies left with the family will be recorded. The MI intervention providers will also record missed visits and all attempts to schedule visits.

#### Measurement of fidelity

All MI sessions are to be audio-recorded, as required for intervention fidelity monitoring by the NIDCR. Ongoing monitoring is to include a review of a random sample of 20% of the intervention recordings, scoring them against the Motivational Interviewing Treatment Integrity (MITI) [29]. Intervention providers will be given feedback on the audited intervention sessions, with special attention to adherence to the MI model and competence in the use of MI. Intervention providers will participate in self-reflection on their adherence and competence as part of the supervision and feedback sessions. For purposes of inter-rater reliability, half of the random sample of sessions selected for review will be rated a second time with the MITI by the MI consultant (10%).

#### Enhanced community services (usual care)

Development of enhanced community services includes text for public service announcements, billboards, recruitment flyers, as well as tri-fold brochures focusing on behavioral risk factors for ECC and oral health topics covered in the MI sessions. Additionally, toothbrushes and toothpaste are given to the participant.

### Primary outcome measure

The dmfs index measures the level of dental caries, the primary outcome measure. The caries experience for a child is expressed as the total number of teeth or surfaces that are decayed (d), missing (m), or filled (f). Study-trained and calibrated licensed dental hygienists blinded to the study condition will conduct visual examinations of the enrolled children’s mouths at aged one, two and three years to determine dmfs, and trained study personnel record the observations. The caries evaluation will be performed annually for three years for several reasons: more frequent contact with the participant may increase the likelihood that the participant will remain in the trial; measuring the outcome at three different time points should potentially increase the likelihood of obtaining useful data, even if the participant misses one or more of the follow-up visits; and multiple assessments of the outcome will increase the statistical power of the final analysis of the trial.

A knee-to-knee examination is completed with the assistance of parents or caregivers. The child’s teeth are brushed to enable adequate visualization of tooth surfaces and a direct light source and mouth mirror are used to systematically evaluate each tooth for the presence of decayed and filled surfaces. Caries detection and measurement criteria as described by Pitts are used to visually evaluate and score lesions [30,31]. The findings are charted in an electronic dental research record called CARIN (Caries Research Instrument) [32], which is used across all three Early Childhood Caries Centers funded by NIDCR.

### Secondary outcome measure

To capture information about parents’ oral health knowledge, attitudes, and behaviors, a parental oral health survey, the Basic Research Factor Questionnaire (BRFQ) was developed. The BRFQ development was a collaborative effort involving the three Collaborating Centers for Early Childhood Caries funded by NIDCR.

Constructs included in the Basic Research Factors Questionnaire

Constructs

Parent and Household Characteristics

Age

Gender

Highest Grade

Employment Status

Ethnicity

Race

Tribal Affiliation

Relationship to Child

Number of Household Members

Annual Household Income

Perceived Adequacy of Income

Child Socio-demographics

Ethnicity

Race

Tribal Affiliation

Age

Child’s Dental Health

Child’s Oral Health Status

Child’s Oral Health Quality of Life

Dental Insurance Coverage

Dental Care Utilization (Preventive and Restorative Care)

Time and Travel Costs (e.g., Missed Work Due to Child Dental Care)

Oral Health Knowledge & Behavior

Oral Health Knowledge

Oral Health Behavior

Oral Health Attitudes

Self-efficacy (Confidence that One Can Engage in Good Oral Health Behavior)

Perceived Importance of Good Oral Health Behavior

Locus of Control Regarding Child’s Oral Health

Perceived Benefits of and Barriers to Good Oral Health Behavior

Perceived Seriousness of Poor Oral Health Outcomes for Child

Perceived Susceptibility of Child to Poor Oral Health Outcomes

Parental Resources, Barriers, and Health Status

Health Literacy

Comorbidities

Parent’s Oral Health Status

Alcohol Use

Distress

Chronic Stress

Perceived Discrimination

Tribal Identity

Sense of Coherence

Social Support

Access to a Working Vehicle

The BRFQ was designed to be administered using an audio computer-assisted self-interviewing (ACASI) system. The survey was programmed in SSI Web from Sawtooth Software [33] and is implemented on encrypted Dell Inspiron Mini laptop computers, Dell, Texas, United States

### Randomization

A stratified blocked randomization was developed by CNOHR biostatistical staff using a random number generator. Randomly selected block sizes of 2 and 4 are used. Blocked randomization of randomly selected sizes is used to prevent the field staff from knowing what treatment the next participant will receive during the recruitment process and to ensure that if the study is stopped at any particular time there will be an approximately equal number of participants in each study arm. Stratification is based on age of mother or caregiver. Randomization is completed over the phone between the field staff enrolling the participant and a staff member at University of Colorado Anschutz Medical Campus. The field staff is required to obtain consent and enroll the subject in the trial prior to seeing the group assignment.

### Blinding

Due to the nature of the interventions, the intervention providers and participants cannot be masked in this study. However, the licensed dental professionals who perform the annual oral health screenings are blinded to group assignment.

### Sample size

Based on the IHS 1999 dental survey of the AI population [1], the mean dmfs for all AI nationally was 10.05 (SD = 16.27) at age two and 12.22 (SD = 19.17) at age three. Assuming a repeated measures analysis with dmfs measured at 3 time points (years one, two and three), an intra-class correlation of 0.50, a 40% reduction in mean dmfs due to the MI intervention, a 90% statistical power using an α = 0.05 2-sided test, and an 80% retention rate, we arrived at a total sample size of 540 mothers and newborns in the two treatment arms (270 per arm). The final target sample size was set at 600 to allow for some potential misspecifications of the statistical assumptions.

### Analyses

The primary outcome measure of caries experience for this study will be the differences in mean dmfs measures for the children in the two treatment arms at ages one, two, and three. Descriptive analyses (frequencies, means, and standard deviations) will be performed first in order to guide the statistical analyses. Longitudinal methods of analysis will be used to examine these repeated and correlated observations within subjects over time. Exploratory analysis will first be done to evaluate the trends in the data in order to determine the most appropriate mean and covariance structures to fit the data. Graphic displays will be used to assess the mean structure for modeling fixed effects, including scatter plots of the response variable versus time, plots of individual subject profiles and plots of mean profiles. Linear mixed models will be used to fit the data. Once a suitable covariance structure has been determined, an appropriate means structure will be determined. The mean structure might be linear or nonlinear over time and each of these will be tested.

Secondary outcomes will include caries patterns, oral health knowledge and behavior and cost analyses. Caries patterns will be analyzed by looking at dmfs components according to specific teeth in the mouth. A cost analysis will be conducted and will relate the cost of the intervention to the results of the study. To facilitate this, data regarding the time required for each aspect of the study will be periodically collected. This will allow for the analysis of personnel costs associated with the intervention. The costs of all enhanced community service efforts will also be readily available for analysis once the study is complete.

## Discussion

As perhaps the first randomized controlled trial of an MI behavioral intervention for ECC in an AI population, the design of this research protocol has involved complex adaptations of behavioral strategies and attention to issues related to acceptability of assessments, interventions, strategies, and procedures.

Major issues for the study were addressed with deliberate community involvement, according to community based participatory research methodology. Presenting issues, seeking feedback and strategizing about solutions as a team empowers and supports the community in seeking sustainable solutions to oral health issues, and establishes trust in the research mechanism. In addition to the biannual Community Advisory Board (CAB) meeting, community members are directly represented as hired staff in the field. The CAB advised our development team on communication norms for the tribe and the design features of all specifically created materials which reflect tribal colors, symbols, and distinguish the materials as being created for this oral health project and this tribe.

This behavioral trial is unusual in two respects. First, it provides enhanced community services to the control group. In this way, we provide some level of community service to everyone participating in the study. Our experience in working with tribal groups made it clear that they would not be comfortable with the absence of services for a control group. Second, the use of MI for behavior change in caries reduction in the intervention is unique in itself and the study will provide a model for aspects of MI implementation, such as desirable characteristics of intervention providers, individual adaptation of the approach, and fidelity assurance.

Challenges to recruitment and retention, which is defined as successful completion of the required study visits, and our approaches to resolving those may also be instructive for future study or implementation of behavioral strategies for oral health. Working on a reservation with a low income, rural and mobile population has its own complexities. Isolated communities and difficult commutes to reach the participant make it harder to recruit and retain participants. Recruitment was not meeting the target in the first year, therefore new recruitment strategies were introduced, such as extending recruitment to nearby areas off the reservation (as the population is mobile) and reaching out to all local programs which provide for maternal and child health. Other strategies include advertisements and public service announcements on local radio stations and flyers posted in public areas and advertisements in area newspapers. As the project increased in number of participants and became known in the local community, we were able to hire more staff from the local community, which has also helped with recruitment efforts.

The weather has been a factor in this study. Severe winter storms in the region may make travel impossible and result in several days of closure of schools, roads, and services annually. Our study follows the local school closure and road closure policy. In all seasons, even when major roads are open, local village roads may not be cleared, thus neither staff nor participants can travel the impassable miles of snow-packed or muddy dirt roads.

Of the stated three primary aims, two have been completed. With the support of the community, culturally specific materials and public service announcements have been developed and are in use with the study participants and for the benefit of all community members through radio, newspaper, and billboard presentations. The MI manual and materials were developed, pilot tested, and in full study use. The remaining primary aim will be met at the conclusion of the study when all participant children who complete the study have their three dental screenings. Data analysis will commence post conclusion of all data collection. The issue of ECC is endemic on the Pine Ridge Reservation and the problem has existed for decades. An effective and parsimonious methodology is needed to stop the generational repetition observed with ECC. This study should provide robust evidence as to the effectiveness of MI in reducing ECC in the first three years of life in this underserved community. If it is effective in reducing the disease, it could provide a methodology to end the pain and discomfort experienced by many AI children. children as they grow from infancy to school age.

### Ethical considerations and approvals

The trial has been approved by the Oglala Sioux Tribe Research Review Board, the Colorado Multiple Institutional Review Board and the Aberdeen Area Indian Health Service Institutional Review Board. This study is reviewed by a number of oversight groups comprised of the National institute of Dental and Craniofacial Research, External Scientific Advisory Committee, Community Advisory Committee, and Data and Safety Monitoring Board.

## Trial status

The trial is currently enrolling participants.

## Abbreviations

ACASI: Audio computer-assisted self-interviewing; AI: American Indians; AN: Alaska Natives; BRFQ: Basic Research Factor Questionnaire; CAB: Community Advisory Board; dmfs: decayed missing filled surfaces; ECC: early childhood caries; ESC: enhanced community services; IHS: Indian Health Service; MI: motivational interviewing.

## Competing interests

All authors declare that they have no competing interests.

## Authors’ contributions

TB: conception and design, data collection and analysis, manuscript writing and final approval of the manuscript. KAF: data collection and analysis, critical revision and final approval of the manuscript. TT: data collection and analysis, manuscript writing and final approval of the manuscript. WGH: conception and design, data collection and analysis, manuscript writing and final approval of the manuscript. AW: conception and design, data collection and analysis, critical revision and final approval of the manuscript. AGB: conception and design, data collection and analysis, critical revision and final approval of the manuscript. JA: conception and design, data collection and analysis, manuscript writing and final approval of the manuscript. All authors read and approved the final manuscript.
